# The Effects of Anesthesia on Adult Hippocampal Neurogenesis

**DOI:** 10.3389/fnins.2020.588356

**Published:** 2020-10-22

**Authors:** Jenny L. Kim, Nicholas E. Bulthuis, Heather A. Cameron

**Affiliations:** Section on Neuroplasticity, National Institute of Mental Health, National Institutes of Health, Bethesda, MD, United States

**Keywords:** adult neurogenesis, hippocampus, isoflurane, propofol, midazolam, dexmedetomidine

## Abstract

In animal studies, prolonged sedation with general anesthetics has resulted in cognitive impairments that can last for days to weeks after exposure. One mechanism by which anesthesia may impair cognition is by decreasing adult hippocampal neurogenesis. Several studies have seen a reduction in cell survival after anesthesia in rodents with most studies focusing on two particularly vulnerable age windows: the neonatal period and old age. However, the extent to which sedation affects neurogenesis in young adults remains unclear. Adult neurogenesis in the dentate gyrus (DG) was analyzed in male and female rats 24 h after a 4-h period of sedation with isoflurane, propofol, midazolam, or dexmedetomidine. Three different cell populations were quantified: cells that were 1 week or 1 month old, labeled with the permanent birthdate markers EdU or BrdU, respectively, and precursor cells, identified by their expression of the endogenous dividing cell marker proliferating cell nuclear antigen (PCNA) at the time of sacrifice. Midazolam and dexmedetomidine reduced cell proliferation in the adult DG in both sexes but had no effect on postmitotic cells. Propofol reduced the number of relatively mature, 28-day old, neurons specifically in female rats and had no effects on younger cells. Isoflurane had no detectable effects on any of the cell populations examined. These findings show no general effect of sedation on adult-born neurons but demonstrate that certain sedatives do have drug-specific and sex-specific effects. The impacts observed on different cell populations predict that any cognitive effects of these sedatives would likely occur at different times, with propofol producing a rapid but short-lived impairment and midazolam and dexmedetomidine altering cognition after a several week delay. Taken together, these studies lend support to the hypothesis that decreased neurogenesis in the young adult DG may mediate the effects of sedation on cognitive function.

## Introduction

In humans, surgery under general anesthesia has been linked to performance deficits in memory and executive function (Monk et al., 2008; Rundshagen, 2014). The specific aspects of surgery that lead to cognitive dysfunction are unknown, but prolonged exposure to general anesthesia has been implicated as a possible contributing factor. These findings raise significant concerns for animal studies in biomedical research as many utilize anesthetics for surgical procedures that may lead to unintended cognitive impairments and/or neurological changes that may confound results if not appropriately accounted for. Preliminary studies show that certain sedative agents can improve (Gertler et al., 2001; Hovaguimian et al., 2018) or worsen (Pandharipande et al., 2006) postoperative outcomes, suggesting that postoperative cognitive dysfunction may be linked to the pharmacodynamics of specific anesthetics rather than surgery or the sedation process itself.

Thus far, most rodent studies have focused on neonates and aged animals, as these two age groups appear to be particularly vulnerable to cognitive impairment following anesthesia exposure. Several reports have found that cognitive impairments can arise in rodents following the administration of anesthesia independent of surgery (Culley et al., 2007; Callaway et al., 2016). More specifically, anesthesia exposure in early life impairs performance on spatial memory and fear conditioning tasks even into adulthood (Jevtovic-Todorovic et al., 2003; Stratmann et al., 2009; Zhu et al., 2010; Kodama et al., 2011; Shen et al., 2013; Landin et al., 2019). In aged rodents, sedation with volatile anesthetics such as isoflurane negatively affects performance on spatial memory, fear conditioning, and odor-reward association tasks (Culley et al., 2003, 2004a,b; Ku et al., 2010; Erasso et al., 2011; Zhang et al., 2012).

The neural mechanisms connecting sedation and cognitive dysfunction remain elusive, but one possible link is a reduction in hippocampal neurogenesis. Similar to the behavioral effects of anesthesia, decreased postnatal generation of granule neurons in the dentate gyrus (DG) of the hippocampus is also associated with impaired learning and memory (Cameron and Glover, 2017) as well as depression (Hill et al., 2015). A significant proportion of granule cell population is generated in the juvenile period (Bayer et al., 1993; Kozareva et al., 2019), so any dysregulation during this period could have large and long-lasting effects on the dentate gyrus. Conversely, in old age, the rate of DG neurogenesis is quite low (Kozareva et al., 2019; Moreno-Jiménez et al., 2019) such that further decrease could leave the DG without sufficient new neurons, making this age group vulnerable as well. The generation of functioning neurons in the DG requires several weeks and involves division of precursor cells, development of a neuronal phenotype, survival of only a fraction of the young neurons, and integration of the new neurons into circuits, and each of these phases can be independently altered by drugs and experiences (Snyder et al., 2009a,b; Soumier et al., 2016). In rodent studies, there is significant evidence that both inhalable sedatives (e.g., isoflurane and sevoflurane) and injectable sedatives (e.g., propofol) can increase cell death and reduce cell proliferation in the neonatal DG (Jevtovic-Todorovic et al., 2003; Stratmann et al., 2009; Broad et al., 2016; Huang et al., 2016; Palanisamy et al., 2017). No changes in cell proliferation or cell death in the DG have been observed following treatment with isoflurane or propofol in aged rats (Ku et al., 2010; Erasso et al., 2011, 2013), but isoflurane decreased survival of 21-day old cells in these animals (Erasso et al., 2013), indicating that the effects of anesthesia on neurogenesis and cognition vary considerably across the lifespan.

Far less attention has been directed toward examining anesthesia-induced cognitive dysfunction in young adults, and the effects that have been observed are often unclear or seemingly inconsistent. Administration of propofol, but not isoflurane, impaired performance of an odor-reward association task in adult rats 2 days after anesthesia (Erasso et al., 2011). Similarly, sevoflurane and isoflurane seem to have opposite effects on behavior in a spatial water maze (Stratmann et al., 2009; Shen et al., 2013). Studies in young adult rodents have found negative effects of sedation on adult neurogenesis (Erasso et al., 2011, 2013; Krzisch et al., 2013; Deng et al., 2014), but many experiments have not found changes in new neurons after sedation, suggesting selective effects of different sedatives (Erasso et al., 2013), at different time points after anesthesia (Erasso et al., 2011; Krzisch et al., 2013), and different phases of neurogenesis (Erasso et al., 2013). Additional studies are required to determine the impact of sedatives on neurogenesis in young adults.

In the current study, we investigated the effects of four different sedatives on generation, maturation, and survival of new neurons in the adult rat DG using endogenous markers of proliferation and neuronal maturation state as well as injectable birth dating markers. Most studies examining the effects of sedation on adult hippocampal neurogenesis have focused on isoflurane and propofol as they are commonly used in clinical and research settings for both deep anesthesia and procedural sedation. While the exact mechanism of action is unknown for these drugs, their sedative effects appear to require activation of GABA_A_ receptors (Garcia et al., 2010). Dexmedetomidine is a potent α_2_-adrenoceptor agonist commonly used for sedation in laboratory animals as well as human patients that reportedly causes less neurocognitive dysfunction and respiratory distress than many sedatives (Gertler et al., 2001). Midazolam, the only water-soluble benzodiazepine, is commonly used for procedural sedation in humans and is increasingly used in animal studies (Olkkola and Ahonen, 2008). We therefore sought to compare the unknown effects on adult neurogenesis of these less investigated two sedatives with those of isoflurane and propofol.

A handful of studies in neonatal rats have observed sex differences in the cognitive effects of sedation (Cabrera et al., 2020). However, the vast majority of investigations into the effects of sedation on cognition and neurogenesis in adults have been done in male rodents, mirroring the predominant use of male subjects in neuroscience and animal research more broadly (Beery and Zucker, 2011). Research examining sex differences in pharmacokinetics and pharmacodynamics has recently gained more attention due to concerns regarding the overdosing of females on several drugs (Soldin and Mattison, 2009; Cahill and Aswad, 2015), underscoring the potential problems of preclinical research limited to one sex. Therefore, the current study included both males and females to assess potential sex differences in anesthesia effects at different stages of adult neurogenesis.

## Materials and Methods

### Animals and Experimental Design

**FIGURE 1:**
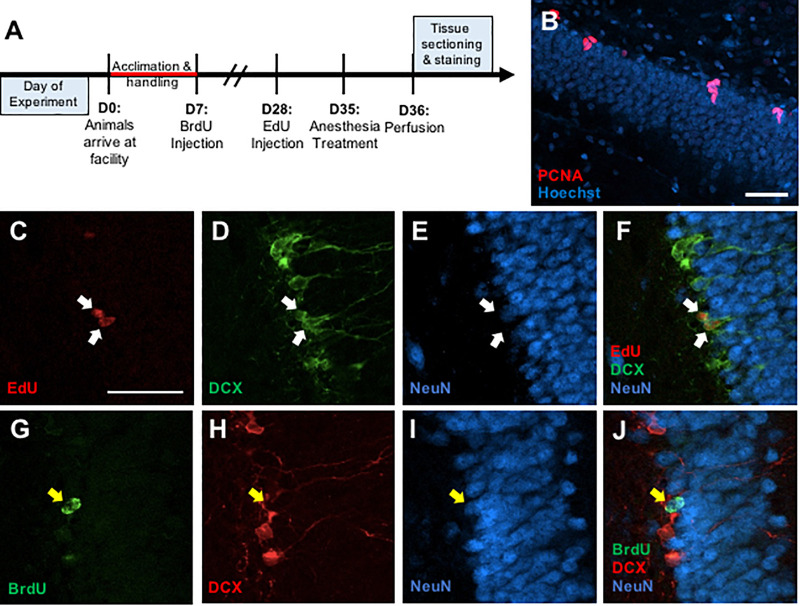
**(A)** Timeline depicting experimental design. **(B)** Representative photomicrograph of coronal section through the adult dentate gyrus granule cell layer showing immunostaining for PCNA (red) counterstained with Hoescht (blue). **(C–F)** Representative confocal images illustrating colocalization of EdU+ cells **(C)** with immature neuronal marker doublecortin (DCX) **(D)**, but no co-expression with mature neuronal marker NeuN **(E)** and a merged image **(F)**. **(G–J)** Representative confocal images showing colocalization of BrdU+ cells **(G)** with NeuN **(H)**, but no colocalization with DCX **(I)**, and a merged image **(J)**. Scale bar = 50 μm.

A total of 42 male and 42 female adult Long-Evans rats (Charles River, Raleigh, NC, United States) were used (*n* = 6–8/treatment/sex). Isoflurane and Propofol were tested in one set of animals, and midazolam and dexmedetomidine were tested in a second set, each with a separate control group. Upon arrival at postnatal day (P) 49, rats were group housed in a reverse 12:12 light/dark cycle (Lights off 9:00 AM) and acclimated to the animal facility and investigator handling for 1 week (Figure 1A). On P56, all rats received a single injection of bromodeoxyuridine (BrdU, Sigma; 200 mg/kg; i.p.). Three weeks later (P77), all rats also received a single injection of ethynyl-deoxyuridine (EdU, Cayman Chemicals or VWR; 50 mg/kg; i.p.).

One week after EdU treatment (1 month after BrdU treatment, P84), animals were randomly assigned to one of the following sedative treatment groups: control, isoflurane, propofol, midazolam, or dexmedetomidine. Control animals received an i.p. injection of 0.9% saline and were immediately returned to their home cages. All other animals were sedated for 4 h. Body temperature was regulated using a heating pad, and breathing patterns were monitored for respiratory distress. Isoflurane was administered with a vaporizer using 4% isoflurane to induce sedation and 1.5–2% isoflurane for maintenance delivered in 2 L/min O_2_ via nose cone. Propofol (90 mg/kg/dose), midazolam (8.0 mg/kg/dose), and dexmedetomidine (0.1 mg/kg/dose) were administered via i.p. injections over a 4-h period with a full dose administered to maintain sedation approximately every hour, or earlier if the rat made any movements suggesting recovery from sedation. After 4 h of sedation, all animals remained on heating pads until they regained consciousness (∼5–20 min) and then placed back into their home cages.

### Tissue Collection and Processing

All animals were perfused the day after anesthesia exposure (P85). Rats were given an overdose of sodium pentobarbital and then transcardially perfused with 4% paraformaldehyde in cold 0.1 M PBS (pH 7.4). Brains were postfixed overnight in 4% paraformaldehyde and then transferred to 20% sucrose solution and stored at 4°C until the brains sank. All brains were coronally sectioned at 40 μm on a sliding microtome into 1:12 series containing the entire DG (bilateral). Sections were stored at 4°C in PBS with 0.1% sodium azide until they were stained.

#### Single-Label PCNA Immunofluorescent Staining

Free-floating sections were rinsed in PBS for 15 min then in 0.1% sodium borohydride for 10 min at room temperature to reduce autofluorescence. After a 30-min PBS rinse, the sections were placed in 0.1 M citric acid (pH = 6.0) for 15 min at 90°C. Sections were rinsed in PBS for 30 min, blocked in PBS containing 0.1% Triton-X-100 and 3% donkey serum for 30 min, and incubated in monoclonal mouse anti-PCNA antibody (1:20,000, sc-56; Santa Cruz Biotechnology, RRID: AB_628110) for 48 h at 4°C. Sections were then rinsed in PBS for 30 min and incubated in a secondary antibody (Alexa Fluor 555 donkey anti-mouse, 1:250, A-31570, Invitrogen, RRID: AB_2536180) for 2 h at room temperature. Sections were washed in PBS for 30 min and then incubated in Hoechst 33258 counterstain (1:1000, Sigma) for 10 min. All sections were washed in PBS for 30 min, mounted onto gelatin-subbed slides, dehydrated, and coverslipped under PVA-DABCO mounting media (Figure 1B).

#### EdU/DCX/NeuN Immunofluorescent Staining

Free-floating sections were rinsed in PBS for 30 min. Then, sections were rinsed in PBS for 30 min and then blocked in PBS containing 0.5% Triton-X-100 and 3% donkey serum for 30 min. Incubation in monoclonal goat anti-DCX (1:200, sc-8066, Santa Cruz Biotechnology) and monoclonal mouse anti-NeuN (1:250, MAB377, Chemicon) occurred for 48 h at 4°C. Sections were then rinsed in PBS for 30 min and incubated in Alexa Fluor 488 donkey anti-goat (1:250, A-11055, Invitrogen) and Alexa Fluor 647 donkey anti-mouse (1:250, A-31571, Invitrogen) for 2 h at room temperature. After a 15-min rinse in PBS, sections were rinsed in a 3% BSA solution for 20 min at RT. Then, sections were incubated in 0.5% Triton-X-100 solution. Following a 10-min rinse in PBS, sections were placed in Click-iT EdU reaction cocktail (C10638, Invitrogen) for 30 min. The sections were given a final rinse in PBS for 20 min and then mounted onto gelatin-subbed slides, dehydrated, and coverslipped under PVA-DABCO mounting media (Figures 1C–F).

#### BrdU/DCX/NeuN Immunofluorescent Staining

Free-floating sections were rinsed in PBS for 30 min. Then, the sections were incubated in 2N HCl for 60 min at 37°C. Afterward, the sections were rinsed in PBS for 30 min and then blocked in PBS containing 0.5% Triton-X-100 and 3% donkey serum for 30 min. The sections placed in monoclonal rat anti-BrdU (1:1000, OBT0030; Accurate, RRID: AB_2313756), monoclonal goat anti-DCX (1:200, sc-8066, Santa Cruz Biotechnology, RRID: AB_2088494), and monoclonal mouse anti-NeuN (1:250, MAB377, Chemicon, RRID: AB_2298772) at 4°C for 48 h. Sections were then rinsed in PBS for 30 min and incubated in Alexa Fluor 488 donkey anti-rat (1:250, A-21208, Invitrogen, RRID: 2535794), Alexa Fluor 555 donkey anti-goat (1:250, A-21432, Invitrogen, RRID: AB_2535853), and Alexa Fluor 647 donkey anti-mouse (1:250, A-31571, Invitrogen, RRID: AB_162542) for 2 h at room temperature. Following a 30-min rinse in PBS, all sections were mounted onto gelatin-subbed slides, dehydrated, and coverslipped under PVA-DABCO mounting media (Figures 1G–J).

### Data Collection

For all analyses, slides were coded to blind the investigator to treatment groups. The single-label counts (i.e., PCNA, BrdU, and EdU) were conducted using an Olympus BX51 microscope under epi-illumination using an UPlanS Apo 40× (0.9 NA) objective. The rostral-caudal extent of the DG was analyzed (10–12 bilateral sections) for each animal. PCNA-positive (+) and EdU+ cells were visualized using a TRITC epifluorescence filter and identified by the presence of a bright orange nuclear stain. BrdU+ cells were visualized by a FITC epifluorescence filter and identified by the bright green nuclear staining. DG area and volume were not controlled for as the single-labeled brain tissue was used to assess total number of PCNA+, EdU+, or BrdU+ cells. The total number of single-labeled cells was determined by summing the number of positively stained cells across all sections analyzed. Immunolabeled sections were analyzed using a Nikon C2+ confocal laser-scanning microscope with a Plan Apochromat 20X (0.75 NA) objective. All confocal images were captured with 488, 561, and 647 nm laser exposures. 50 BrdU+ and 50 EdU+ cells in the DG of each animal were analyzed using a z-stack orthogonal viewer to verify colocalization of BrdU and EdU with DCX or NeuN. The first 25 cells identified from dorsal sections and first 25 cells from ventral sections were chosen. Z-stacks were created at a 0.85 μm intervals throughout the 40 μm section to confirm double-labeling of BrdU-ir cells.

### Statistical Analysis

To examine group differences in total numbers of cells labeled with PCNA, BrdU, and EdU, a two-way ANOVA was run using treatment group (anesthetic or control) and sex as factors. For the immunofluorescence analyses, a two-way ANOVA (treatment group by sex) was used to determine whether the% colocalization of BrdU/DCX, BrdU/NeuN, EdU/DCX, and EdU/NeuN differed between treatment groups. For all analyses, a significance value of *p* < 0.05 was used and significant results were followed by a Tukey’s *post hoc* test.

## Results

### Midazolam and Dexmedetomidine Reduce Cell Proliferation in the DG of Adult Rats

**FIGURE 2:**
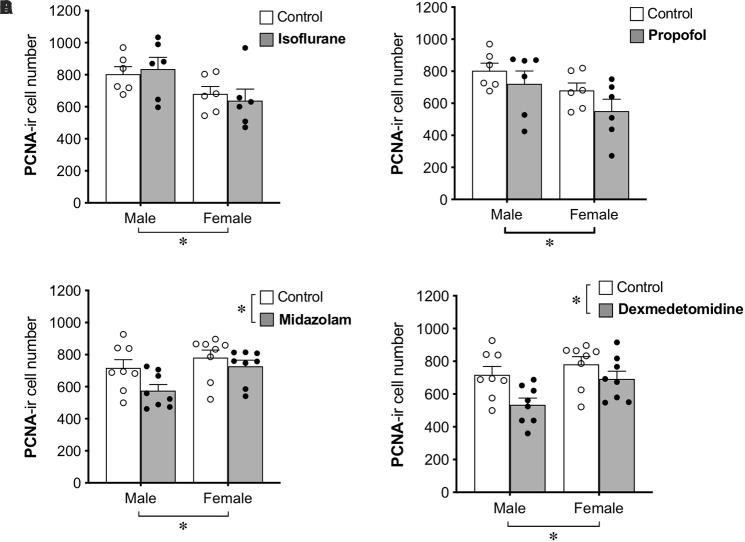
Administration of midazolam and dexmedetomidine reduces cell proliferation in dentate gyrus of adult rats. Neither isoflurane **(A)** nor propofol **(B)** significantly affected the number of PCNA+ cells in the DG of either sex. Both midazolam **(C)** and dexmedetomidine **(D)** significantly reduced cell proliferation in the DG. There were no significant sex × treatment interactions for any drug, but main effects of sex in different directions and a lack of sex effect in control animals suggest possible undetected interactions. ^∗^*p* < 0.05. Data are represented as mean ± SEM.

Group differences in cell proliferation were assessed by counting the total number of PCNA+ cells in the DG (Figure 2). No main effect of treatment was found for propofol treatment, but a main effect of sex difference was detected, with female rats showing ∼20% fewer PCNA+ cells compared to males. Similarly, no main effect of isoflurane treatment was observed, but female rats had significantly lower PCNA+ cell counts than males.

Treatment with midazolam significantly decreased PCNA+ cell number by 13%, and treatment with dexmedetomidine significantly decreased PCNA+ cell number by 17%. Both of these analyses also showed main effects of sex with male rats having fewer PCNA+ cells than females. Neither analysis demonstrated a significant interaction between sex and treatment, but no sex difference was observed in control animals (Table 1), suggesting that the sex difference may have been driven by greater reduction of cell proliferation by sedation in males compared to females.

**FIGURE 3:**
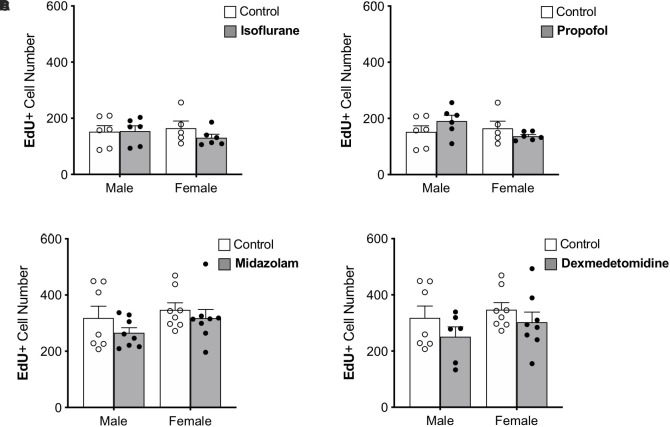
Treatment with anesthesia does not change the number of 7-day-old neurons in the dentate gyrus of adult male and female rats. No significant main effects of treatment or sex was observed when rats were administered **(A)** isoflurane, **(B)** propofol, **(C)** midazolam, or **(D)** dexmedetomidine. Data are represented as mean ± SEM.

**TABLE 1:** Statistical analyses.

	Referred to in	Type of test	Mean (SEM)	Statistical data
(a)	Figure 2A (PCNA)	Two-Way ANOVA	Control (*M*) = 803.3(46.9) Control (*F*) = 680.4 (46.8) Isoflurane (*M*) = 835.5 (72.9) Isoflurane (*F*) = 638.7 (71.8)	Sex: *F*_(__1_,_20__)_ = 6.888, ***p* = 0.016** Treatment: *F*_(__1_,_20__)_ = 0.006, *p* = 0.940 Sex × Treatment: *F*_(__1_,_20__)_ = 0.365, *p* = 0.553
(b)	Figure 2B (PCNA)	Two-Way ANOVA	Control (*M*) = 803.3 (46.9) Control (*F*) = 680.4 (46.8) Propofol (*M*) = 721.0 (80.4) Propofol (*F*) = 551.5 (73.6)	Sex: *F*_(__1_,_20__)_ = 5.262, ***p* = 0.033** Treatment: *F*_(__1_,_20__)_ = 2.735, *p* = 0.114 Sex × Treatment: *F*_(__1_,_20__)_ = 0.132, *p* = 0.720
(c)	Figure 2C (PCNA)	Two-Way ANOVA	Control (*M*) = 717.9 (50.7) Control (*F*) = 781.1 (47.8) Midazolam (*M*) = 575.3 (38.4) Midazolam (*F*) = 727.9 (37.6)	Sex: *F*_(__1_,_28__)_ = 6.023, ***p* = 0.021** Treatment: *F*_(__1_,_28__)_ = 4.959, ***p* = 0.034** Sex × Treatment: *F*_(__1_,_28__)_ = 1.032, *p* = 0.318
(d)	Figure 2D (PCNA)	Two-Way ANOVA	Control (*M*) = 717.9 (50.7) Control (*F*) = 781.1 (47.8) Dexmed (*M*) = 548.7 (44.7) Dexmed (*F*) = 692.4 (47.3)	Sex: *F*_(__1_,_28__)_ = 4.641, ***p* = 0.040** Treatment: *F*_(__1_,_28__)_ = 7.211, ***p* = 0.012** Sex × Treatment: *F*_(__1_,_28__)_ = 0.701, *p* = 0.410
(e)	Figure 3A (EdU)	Two-Way ANOVA	Control (*M*) = 152.0 (21.7) Control (*F*) = 164.8 (25.4) Isoflurane (*M*) = 154.2 (19.1) Isoflurane (*F*) = 130.8 (12.3)	Sex: *F*_(__1_,_19__)_ = 1.110, *p* = 0.305 Treatment: *F*_(__1_,_19__)_ = 0.074, *p* = 0.789 Sex × Treatment: *F*_(__1_,_19__)_ = 2.936, *p* = 0.103
(f)	Figure 3B (EdU)	Two-Way ANOVA	Control (*M*) = 152.0 (21.7) Control (*F*) = 164.8 (25.4) Propofol (*M*) = 190.5 (20.6) Propofol (*F*) = 136.8 (6.0)	Sex: *F*_(__1_,_19__)_ = 0.071, *p* = 0.793 Treatment: *F*_(__1_,_19__)_ = 0.644, *p* = 0.432 Sex × Treatment: *F*_(__1_,_19__)_ = 0.832, *p* = 0.373
(g)	Figure 3C (EdU)	Two-Way ANOVA	Control (*M*) = 318.0 (42.2) Control (*F*) = 347.3 (24.9) Midazolam (*M*) = 265.5 (18.3) Midazolam (*F*) = 317.6 (31.4)	Sex: *F*_(__1_,_27__)_ = 1.883, *p* = 0.181 Treatment: *F*_(__1_,_27__)_ = 1.918, *p* = 0.177 Sex × Treatment: *F*_(__1_,_27__)_ = 0.149, *p* = 0.703
(h)	Figure 3D (EdU)	Two-Way ANOVA	Control (*M*) = 318.0 (42.2) Control (*F*) = 347.3 (24.9) Dexmed (*M*) = 251.2 (34.9) Dexmed (*F*) = 302.8 (35.9)	Sex: *F*_(__1_,_25__)_ = 1.339, *p* = 0.258 Treatment: *F*_(__1_,_25__)_ = 2.540, *p* = 0.124 Sex × Treatment: *F*_(__1_,_25__)_ = 0.102, *p* = 0.752
(i)	Figure 4A (BrdU)	Two-Way ANOVA	Control (*M*) = 641.83 (45.7) Control (*F*) = 564.7 (44.4) Isoflurane (*M*) = 592.2 (39.6) Isoflurane (*F*) = 463.8 (44.4)	Sex: *F*_(__1_,_20__)_ = 6.639, ***p* = 0.018** Treatment: *F*_(__1_,_20__)_ = 3.561, *p* = 0.074 Sex × Treatment: *F*_(__1_,_20__)_ = 0.412, *p* = 0.528
(j)	Figure 4B (BrdU)	Two-Way ANOVA	Control (*M*) = 641.83 (45.7) Control (*F*) = 564.7 (44.4) Propofol (*M*) = 651.7 (44.3) Propofol (*F*) = 371.3 (43.5)	Sex: *F*_(__1_,_20__)_ = 19.133, ***p* = 0.000** Treatment: *F*_(__1_,_20__)_ = 5.041, ***p* = 0.036** Sex × Treatment: *F*_(__1_,_20__)_ = 6.179, ***p* = 0.022**
(k)	Figure 4C (BrdU)	Two-Way ANOVA	Control (*M*) = 580.5 (78.3) Control (*F*) = 536.9 (59.7) Midazolam (*M*) = 642.4 (75.3) Midazolam (*F*) = 576.8 (21.3)	Sex: *F*_(__1_,_27__)_ = 0.810, *p* = 0.377 Treatment: *F*_(__1_,_27__)_ = 0.702, *p* = 0.410 Sex × Treatment: *F*_(__1_,_27__)_ = 0.031, *p* = 0.863
(l)	Figure 4D (BrdU)	Two-Way ANOVA	Control (*M*) = 580.5 (78.3) Control (*F*) = 536.9 (59.7) Dexmed (*M*) = 613.4 (70.2) Dexmed (*F*) = 520.1 (39.6)	Sex: *F*_(__1_,_25__)_ = 1.235, *p* = 0.277 Treatment: *F*_(__1_,_25__)_ = 0.017, *p* = 0.897 Sex × Treatment: *F*_(__1_,_25__)_ = 0.163, *p* = 0.690
(m)	Text section “Midazolam and Dexmedetomidine Reduce Cell Proliferation in the DG of Adult Rats” (control comparison PCNA)	Two-Way ANOVA	Control 1 (*M*) = 803.3 (46.9) Control 1 (*F*) = 680.2 (46.8) Control 2 (*M*) = 717.9 (50.7) Control 2 (*F*) = 781.1 (47.8)	Sex: *F*_(__1_,_24__)_ = 0.366, *p* = 0.551 Group: *F*_(__1_,_24__)_ = 0.025, *p* = 0.877 Sex × Treatment: *F*_(__1_,_24__)_ = 3.542, *p* = 0.072
(n)	Text Section “None of the Sedatives Affected 7-Day-Old Neurons” (control comparison EdU)	Two-Way ANOVA	Control 1 (*M*) = 152.0 (21.8) Control 1 (*F*) = 164.8 (25.4) Control 2 (*M*) = 318.0 (42.2) Control 2 (*F*) = 347.3 (25.0)	Sex: *F*_(__1_,_22__)_ = 0.449, *p* = 0.510 Group: *F*_(__1_,_22__)_ = 30.810, ***p* < 0.0001** Sex × Treatment: *F*_(__1_,_22__)_ = 0.068, *p* = 0.796
(o)	Text section “None of the Sedatives Affected 7-Day-Old Neurons” (control comparison BrdU)	Two-Way ANOVA	Control 1 (*M*) = 641.8 (45.7) Control 1 (*F*) = 564.7 (27.2) Control 2 (*M*) = 580.5 (78.3) Control 2 (*F*) = 536.9 (59.7)	Sex: *F*_(__1_,_22__)_ = 1.097, *p* = 0.306 Group: *F*_(__1_,_22__)_ = 0.597, *p* = 0.448 Sex × Treatment: *F*_(__1_,_22__)_ = 0.085, *p* = 0.774
(p)	Text section “Propofol Reduces the Number of Mature Neurons in the DG of Female, But Not Male, Adult Rats” (% colocalization EdU/DCX)	Two-Way ANOVA	Control (*M*) = 99.3 (0.4) Control (*F*) = 98.0 (0.9) Isoflurane (*M*) = 98.3 (0.8) Isoflurane (*F*) = 98.0 (0.7)	Sex: *F*_(__1_,_19__)_ = 1.331, *p* = 0.263 Treatment: *F*_(__1_,_19__)_ = 0.479, *p* = 0.497 Sex × Treatment: *F*_(__1_,_19__)_ = 0.479, *p* = 0.497
(o)	Text section “Propofol Reduces the Number of Mature Neurons in the DG of Female, But Not Male, Adult Rats” (% colocalization EdU/DCX)	Two-Way ANOVA	Control (*M*) = 99.3 (0.4) Control (*F*) = 98.0 (0.9) Propofol (*M*) = 97.0 (0.9) Propofol (*F*) = 98.3 (1.3)	Sex: *F*_(__1_,_19__)_ = 0.000, *p* = 1.000 Treatment: *F*_(__1_,_19__)_ = 1.147, *p* = 0.298 Sex × Treatment: *F*_(__1_,_19__)_ = 2.039, *p* = 0.170
(p)	Text section “Propofol Reduces the Number of Mature Neurons in the DG of Female, But Not Male, Adult Rats” (% colocalization EdU/DCX)	Two-Way ANOVA	Control (*M*) = 97.7 (0.9) Control (*F*) = 96.8 (1.0) Midazolam(*M*) = 96.0 (0.9) Midazolam (*F*) = 97.8 (1.1)	Sex: *F*_(__1_,_27__)_ = 0.0.156, *p* = 0.696 Treatment: *F*_(__1_,_27__)_ = 0.129, *p* = 0.722 Sex × Treatment: *F*_(__1_,_27__)_ = 1.861, *p* = 0.184
(q)	Text section “Propofol Reduces the Number of Mature Neurons in the DG of Female, But Not Male, Adult Rats” (% colocalization EdU/DCX)	Two-Way ANOVA	Control (*M*) = 97.7 (0.9) Control (*F*) = 96.9 (1.0) Dexmed (*M*) = 95.2 (1.4) Dexmed (*F*) = 97.3 (1.0)	Sex: *F*_(__1_,_25__)_ = 0.270, *p* = 0.608 Treatment: *F*_(__1_,_25__)_ = 0.904, *p* = 0.351 Sex × Treatment: *F*_(__1_,_25__)_ = 2.002, *p* = 0.169
(r)	Text section “Propofol Reduces the Number of Mature Neurons in the DG of Female, But Not Male, Adult Rats” (% colocalization EdU/NeuN)	Two-Way ANOVA	Control (*M*) = 0.3 (0.3) Control (*F*) = 0.4 (0.4) Isoflurane (*M*) = 0.3 (0.3) Isoflurane (*F*) = 0.0 (0.0)	Sex: *F*_(__1_,_19__)_ = 0.440, *p* = 0.515 Treatment: *F*_(__1_,_19__)_ = 0.440, *p* = 0.515 Sex × Treatment: *F*_(__1_,_19__)_ = 0.196, *p* = 0.663
(s)	Text section “Propofol Reduces the Number of Mature Neurons in the DG of Female, But Not Male, Adult Rats” (% colocalization EdU/NeuN)	Two-Way ANOVA	Control (*M*) = 0.3 (0.3) Control (*F*) = 0.4 (0.4) Propofol (*M*) = 0.7 (0.4) Propofol (*F*) = 0.0 (0.0)	Sex: *F*_(__1_,_19__)_ = 0.000, *p* = 1.000 Treatment: *F*_(__1_,_19__)_ = 1.147, *p* = 0.298 Sex × Treatment: *F*_(__1_,_19__)_ = 2.039, *p* = 0.170
(t)	Text section “Propofol Reduces the Number of Mature Neurons in the DG of Female, But Not Male, Adult Rats” (% colocalization EdU/NeuN)	Two-Way ANOVA	Control (*M*) = 0.0 (0.0) Control (*F*) = 0.5 (0.3) Midazolam(*M*) = 0.0 (0.0) Midazolam (*F*) = 0.4 (0.4)	Sex: *F*_(__1_,_27__)_ = 2.877, *p* = 0.101 Treatment: *F*_(__1_,_27__)_ = 0.059, *p* = 0.810 Sex × Treatment: *F*_(__1_,_27__)_ = 0.059, *p* = 0.810
(u)	Text section “Propofol Reduces the Number of Mature Neurons in the DG of Female, But Not Male, Adult Rats” (% colocalization EdU/NeuN)	Two-Way ANOVA	Control (*M*) = 0.0 (0.0) Control (*F*) = 0.5 (0.3) Dexmed (*M*) = 0.0 (0.0) Dexmed (*F*) = 0.5 (0.3)	Sex: *F*_(__1_,_25__)_ = 3.723, *p* = 0.065 Treatment: *F*_(__1_,_25__)_ = 0.000, *p* = 1.000 Sex × Treatment: *F*_(__1_,_25__)_ = 0.000, *p* = 1.000
(v)	Text section “Propofol Reduces the Number of Mature Neurons in the DG of Female, But Not Male, Adult Rats” (% colocalization BrdU/DCX)	Two-Way ANOVA	Control (*M*) = 18.3 (2.2) Control (*F*) = 12.3 (2.9) Isoflurane (*M*) = 20.3(2.3) Isoflurane (*F*) = 19.7 (3.7)	Sex: *F*_(__1_,_20__)_ = 1.370, *p* = 0.256 Treatment: *F*_(__1_,_20__)_ = 2.685, *p* = 0.117 Sex × Treatment: *F*_(__1_,_20__)_ = 0.877, *p* = 0.360
(w)	Text section “Propofol Reduces the Number of Mature Neurons in the DG of Female, But Not Male, Adult Rats” (% colocalization BrdU/DCX)	Two-Way ANOVA	Control (*M*) = 18.3 (2.2) Control (*F*) = 12.3 (2.9) Propofol (*M*) = 18.0 (0.9) Propofol (*F*) = 20.7 (2.7)	Sex: *F*_(__1_,_20__)_ = 0.524, *p* = 0.477 Treatment: *F*_(__1_,_20__)_ = 3.019, *p* = 0.098 Sex × Treatment: *F*_(__1_,_20__)_ = 3.543, *p* = 0.074
(x)	Text section “Propofol Reduces the Number of Mature Neurons in the DG of Female, But Not Male, Adult Rats” (% colocalization BrdU/DCX)	Two-Way ANOVA	Control (*M*) = 11.3 (1.2) Control (*F*) = 13.7 (1.1) Midazolam(*M*) = 12.9 (2.1) Midazolam (*F*) = 12.7 (1.6)	Sex: *F*_(__1_,_27__)_ = 0.543, *p* = 0.468 Treatment: *F*_(__1_,_27__)_ = 0.028, *p* = 0.869 Sex × Treatment: *F*_(__1_,_27__)_ = 0.648, *p* = 0.428
(y)	Text section “Propofol Reduces the Number of Mature Neurons in the DG of Female, But Not Male, Adult Rats” (% colocalization BrdU/DCX)	Two-Way ANOVA	Control (*M*) = 11.3 (1.2) Control (*F*) = 13.8 (1.1) Dexmed (*M*) = 12.5 (1.3) Dexmed (*F*) = 13.8 (1.3)	Sex: *F*_(__1_,_25__)_ = 2.107, *p* = 0.159 Treatment: *F*_(__1_,_25__)_ = 0.250, *p* = 0.622 Sex × Treatment: *F*_(__1_,_25__)_ = 0.250, *p* = 0.622
(z)	Text section “Propofol Reduces the Number of Mature Neurons in the DG of Female, But Not Male, Adult Rats” (% colocalization BrdU/NeuN)	Two-Way ANOVA	Control (*M*) = 75.3 (3.3) Control (*F*) = 79.0 (4.1) Isoflurane (*M*) = 73.7 (2.6) Isoflurane (*F*) = 73.7 (4.5)	Sex: *F*_(__1_,_20__)_ = 0.247, *p* = 0.625 Treatment: *F*_(__1_,_20__)_ = 0.900, *p* = 0.354 Sex × Treatment: *F*_(__1_,_20__)_ = 0.247, *p* = 0.625
(aa)	Text section “Propofol Reduces the Number of Mature Neurons in the DG of Female, But Not Male, Adult Rats” (% colocalization BrdU/NeuN)	Two-Way ANOVA	Control (*M*) = 75.3 (3.3) Control (*F*) = 79.0 (4.1) Propofol (*M*) = 75.0 (0.9) Propofol (*F*) = 74.7 (3.1)	Sex: *F*_(__1_,_20__)_ = 0.292, *p* = 0.595 Treatment: *F*_(__1_,_20__)_ = 0.572, *p* = 0.458 Sex × Treatment: *F*_(__1_,_20__)_ = 0.420, *p* = 0.524
(bb)	Text section “Propofol Reduces the Number of Mature Neurons in the DG of Female, But Not Male, Adult Rats” (% colocalization BrdU/NeuN)	Two-Way ANOVA	Control (*M*) = 82.7 (1.0) Control (*F*) = 79.3 (1.4) Midazolam (*M*) = 81.4 (1.2) Midazolam (*F*) = 81.0 (1.9)	Sex: *F*_(__1_,_25__)_ = 1.709, *p* = 0.203 Treatment: *F*_(__1_,_25__)_ = 0.030, *p* = 0.863 Sex × Treatment: *F*_(__1_,_25__)_ = 1.032, *p* = 0.319
(cc)	Text section “Propofol Reduces the Number of Mature Neurons in the DG of Female, But Not Male, Adult Rats” (% colocalization BrdU/NeuN)	Two-Way ANOVA	Control (*M*) = 82.7 (1.0) Control (*F*) = 79.3 (1.4) Dexmed (*M*) = 81.4 (1.7) Dexmed (*F*) = 80.0 (1.1)	Sex: *F*_(__1_,_25__)_ = 3.335, *p* = 0.080 Treatment: *F*_(__1_,_25__)_ = 0.034, *p* = 0.856 Sex × Treatment: *F*_(__1_,_25__)_ = 0.561, *p* = 0.461

*Bold text indicates statistically significant difference (p < 0.05).*

### None of the Sedatives Affected 7-Day-Old Neurons

Immature neurons were quantified by counting the total number of DG cells labeled with EdU administered 7 days prior to sedation (Figure 3). Approximately twice as many EdU+ cells were counted in the first set of control rats (used for propofol and isoflurane experiments) than in the second (used for midazolam and dexmedetomidine experiments); this difference that may be related to the different sources of EdU used for the two groups. However, as each drug-treated group was compared with animals injected at the same time with the same EdU solution, the differences seen in controls should not affect comparisons. No other differences were found between control groups (Table 1).

None of the sedatives affected the number of EdU+ cells in the DG. In addition, no sex differences were observed in any analysis (Table 1).

The proportions of EdU+ cells that expressed DCX, an immature neuron marker, and NeuN, a mature neuronal marker were assessed. More than 95% of EdU+ cells colocalized with DCX and less than 1% co-expressed NeuN in each group, suggesting that nearly all 7-day old cells in the DG were immature neurons as expected. No significant differences were observed in the proportions of EdU+ cells co-expressing either marker, providing no indication that the rate of maturation is affected by sex or sedatives.

### Propofol Reduces the Number of Mature Neurons in the DG of Female, but Not Male, Adult Rats

**FIGURE 4:**
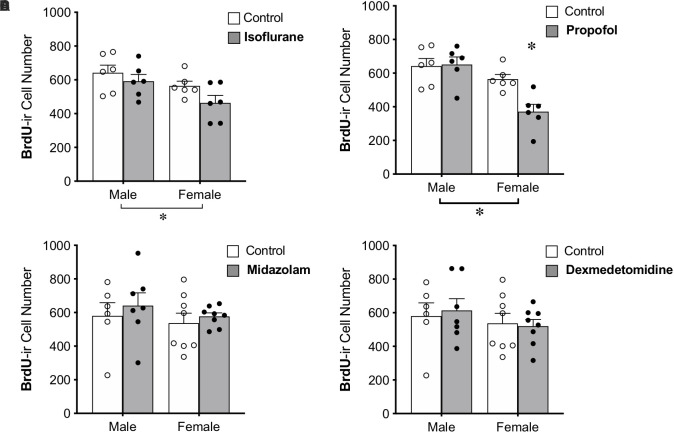
Administration of propofol reduced the number of 4-week-old neurons in the dentate gyrus in adult female, but not male, rats. **(A)** No significant main effect of treatment was observed when rats were administered isoflurane. **(B)** Female rats given propofol has significantly fewer BrdU+ cells in the DG than males treated with propofol and both male and female controls. Overall, female rats had significantly fewer BrdU+ cells than males, regardless of treatment with propofol or isoflurane. Two-way ANOVA followed by Tukey’s *post hoc* test between all groups ^∗^*p* < 0.05. There was no significant main effect of sex or treatment or interaction when male and female rats were given **(C)** midazolam or **(D)** dexmedetomidine. Data are represented as mean ± SEM.

BrdU was administered to all animals 28 days prior to anesthesia exposure to label adult-born neurons that are likely to be mature enough to be integrated into functional neural circuits and able to affect behavior (Snyder et al., 2009b; Gonçalves et al., 2016; Weeden et al., 2019), though new neurons continue to mature morphologically for several more weeks (John et al., 2020). Treatment with propofol showed a sex by treatment interaction, in which propofol decreased BrdU+ cell counts in female rats (by 34%) but not in male rats (Table 1 and Figure 4B). This group also showed significant main effects of sex and treatment (Table 1).

Administration of isoflurane did not significantly affect the number of BrdU+ cells, but a sex difference was identified with females having 17% fewer BrdU+ cells in the DG than males (Table 1 and Figure 4A). No main effects of treatment or sex were detected for midazolam or dexmedetomidine (Figures 4C,D).

Immunofluorescent labeling was used to examine the proportion of BrdU+ cells co-expressing DCX or NeuN in the DG. On average, 11–21% of BrdU+ cells colocalized with DCX and 74–83% of BrdU+ cells co-expressed NeuN in each group, suggesting that most of the cells had matured by this timepoint. No significant differences were found in the colocalization of BrdU+ cells with DCX or NeuN across groups, providing no evidence for effects of sex or sedation on maturation (Table 1).

## Discussion

The current study examined the effects of four different sedatives on adult neurogenesis in the DG of adult male and female rats. By using two cell birthdate markers, BrdU and EdU, and an endogenous cell proliferation marker, PCNA, we were able to assess the effects of anesthesia on three distinct cell populations and found that cell age affects vulnerability to the pharmacological effects of sedatives. Specifically: (1) prolonged treatment with midazolam and dexmedetomidine, but not isoflurane or propofol, reduced cell proliferation in the DG of male and female adult rats 24 h after sedation; (2) none of the tested sedatives affected the number of immature neurons (7 days old) in the DG of either sex; and (3) prolonged sedation with propofol reduced the number of relatively mature adult-born neurons (28 days old) specifically in female rats. These results demonstrate that anesthesia can influence adult hippocampal neurogenesis in a sex-, maturation stage-, and drug-dependent manner.

### Neuropharmacological Mechanisms of Sedatives

As they are currently understood, the specific pharmacological actions of the sedatives used in this study cannot readily explain their differential effects on adult neurogenesis. Though isoflurane, propofol, and midazolam act at different sites on the GABA_A_ receptor (Olkkola and Ahonen, 2008; Vanlersberghe and Camu, 2008) they are all positive allosteric modulators (Schüttler and Schwilden, 2008). Yet only propofol detectably affected survival of maturing granule cells. This effect of propofol on neurogenesis could potentially occur via reported secondary sites of action such as cannabinoid receptors or TRP channels (Patel et al., 2003; Jin et al., 2004; Vennekens et al., 2012; Nishimoto et al., 2015; Rodrigues et al., 2017).

Our investigation of cell proliferation found that dexmedetomidine and midazolam, which act on completely different receptors, both reduced neuronal precursor proliferation. However, despite their disparate direct actions, both drugs are believed to produce their sedative effects through actions, direct or indirect, on GABAergic activity. Midazolam, a benzodiazepine, acts directly at the benzodiazepine site on the GABA_A_ receptor to increase channel opening. An *in vitro* study found that midazolam reduced proliferation of isolated neural stem cells (Zhao et al., 2012), suggesting that it may act directly on granule cell precursors to inhibit proliferation. Dexmedetomidine, in contrast, is a highly specific α2-adrenergic agonist. Its activation of adrenergic receptors is believed to produce sedative effects by inhibiting the release of norepinephrine, which then disinhibits downstream GABAergic neurons in the ventrolateral preoptic nucleus of the hypothalamus (Olkkola and Ahonen, 2008), or other recently identified anesthesia-inducing cell populations (Sukhotinsky et al., 2016; Jiang-Xie et al., 2019), resulting in sedation. A similar circuit involving noradrenergic inhibition of GABAergic interneurons is present in the DG as well (Harley, 2007) and could potentially drive changes in cell proliferation. Interestingly, depletion of norepinephrine using a noradrenergic neurotoxin decreases cell proliferation in the hippocampus of adult male rats (Kulkarni et al., 2002), similar to the effects of dexmedetomidine observed in this study.

Midazolam and dexmedetomidine may decrease neuronal precursor proliferation through a common pathway involving GABAergic signaling, but this would not explain why isoflurane and propofol do not have similar anti-proliferative effects. An alternative explanation relates to secondary aspects of the sedation experience. These light sedatives leave human patients with increased awareness during sedation (McQuaid and Laine, 2008; Schüttler and Schwilden, 2008; Valentine et al., 2012; Scott-Warren and Sebastian, 2016) and slower or poorer recovery post-sedation (Kong et al., 1989; Garrity et al., 2014), particularly when used to maintain sleep (Olkkola and Ahonen, 2008). This prolonged twilight state could be stressful for the animal, leading to inhibition of precursor proliferation (Glasper et al., 2012; Koutmani and Karalis, 2015).

### Effect of Sedatives on Cell Proliferation

We found no change in cell proliferation 1 day after isoflurane or propofol. This is consistent with previous studies showing no significant changes at this same time point after isoflurane (Stratmann et al., 2009; Zhu et al., 2010; Erasso et al., 2013) or propofol (Erasso et al., 2013). Cell proliferation was also unaffected by these sedatives when given during sedation in one study (Tung et al., 2008) but was decreased by isoflurane in another (Stratmann et al., 2009). Intriguingly, two studies found that cell proliferation is increased 4 days after isoflurane, and may remain increased for as long as 16 days (Stratmann et al., 2009, 2010), suggesting a rebound and possible net increase in neuron production after isoflurane treatment.

Few studies have examined the effects of light sedatives such as midazolam and dexmedetomidine on neurogenesis. One study found reduced cell proliferation in the DG of neonatal rats following midazolam treatment (Giri et al., 2018), consistent with what we observed here in the adult rat. Previous work on dexmedetomidine found that this sedative had no effect on cell proliferation during the sedation period (Tung et al., 2008), consistent with the time-dependent effects of isoflurane described above. Taken together, these results suggest that cell proliferation is inhibited for a period of time after recovery from sedation and that midazolam and dexmedetomidine are more likely to produce this inhibitory effect. As discussed earlier, it is possible that increased stress levels contribute to the disparate findings on cell proliferation observed between light and deep sedatives.

### Differential Effects of Sedatives on Survival at Distinct Stages of Adult Neurogenesis

In addition to its effects on cell proliferation, we found that sedation can inhibit survival of adult-born neurons in the DG. In adulthood, most new cells die before functional integration into existing neural circuits (Dayer et al., 2003; Snyder et al., 2009a). Survival of the new neurons is believed to depend on their incorporation into active circuits and the resulting high rate of synaptic input and uptake of neurotrophic factors (Cao et al., 2004; Sairanen et al., 2005; Scharfman et al., 2005; Ge et al., 2008). In neonatal rats, propofol exposure disrupts the signaling pathway of brain derived neurotrophic factor (BDNF), a neurotrophic factor important for neuronal survival (Zhong et al., 2018; Zhou et al., 2018).

We observed no effect on survival of newly-generated cells from midazolam or dexmedetomidine, the two sedatives that affect cell proliferation. This is the first study to examine the role of either of these sedatives on new neuron survival, though a previous study showed no effect of other benzodiazepines on survival of 4- to 14-day-old granule cells (Karten et al., 2006). We also found no effect of isoflurane on survival of 7- or 28-day old cells, consistent with an earlier study of isoflurane effects in male rats that showed no changes in survival of 8-day old or 21-day old neurons (Erasso et al., 2013). However, another study found granule cell death induced by isoflurane that peaked when cells were about 2 weeks old (Hofacer et al., 2013), suggesting that the effect of isoflurane may be limited to a very specific time window that was not captured in the current study. Propofol reduced the number of 28-day old, but not 7-day-old neurons in the DG of female rats in the current study. A previous study found that propofol decreased the survival of 17-day-old, but not 11-day-old, neurons in mixed male and female mice (Krzisch et al., 2013), hinting at the possibility that the window of sensitivity may be longer for propofol than for isoflurane and for cells in females than for cells in males. Together, these findings support the idea that vulnerability to anesthesia-induced neurotoxicity may depend more on the age of cells rather than the age of the subject (Vutskits and Xie, 2016) but is both sex- and drug-dependent.

### Sex Differences in Adult Neurogenesis Following Sedation

In the isoflurane and propofol analyses, we observed a main effect of sex, with female rats having lower levels of cell proliferation than males in the DG. In the midazolam and dexmedetomidine analyses, we found the opposite effect of sex, with females having higher rates of cell proliferation than males, as seen in significant main effects of sex. However, analyzing the control groups alone showed no effect of sex, suggesting that the sex differences in both directions may have resulted from treatment with sedatives, despite a lack of significant sex by drug interactions. In the literature, reports on baseline sex differences in cell proliferation vary depending on specific experimental parameters. Several studies assessing cell proliferation, specifically, in the adult dentate gyrus have found no sex differences under baseline conditions in laboratory rats or mice, or wild-caught voles, chipmunks or squirrels (Barker et al., 2005; Barha et al., 2011; Spritzer et al., 2017; Tzeng et al., 2017). Previous studies have found an estrogen- and estrous cycle-dependent enhancement of new neurons in female rats and voles 1–7 days after labeling (Galea and McEwen, 1999; Tanapat et al., 1999), suggesting a sex difference in the survival time course for new neurons. However, this difference is no longer apparent after 28 days (Galea and McEwen, 1999; Tanapat et al., 1999). The lack of sex difference in baseline numbers of 7- or 28-day-old cells labeled with EdU or BrdU in the current study is consistent with this and suggests that the sex difference in early cell survival is no longer detectable after a week.

We identified a sex difference in the effects of propofol, which reduced survival of 28-day old neurons in the adult hippocampus in females but not males. It is unclear how propofol might affect males and females differently, but it could potentially be related to differences in the maturation rate of new granule neurons. Death of adult-born granule cells is normally undetectable after 28 days in male rats (Dayer et al., 2003), but a recent study in adult rats showed that maturation and attrition of new cells in the DG is prolonged in females relative to males (Yagi et al., 2019). A change in the maturation time course could shift or lengthen the window of susceptibility to a propofol-induced activity decrease in new neurons in females relative to males. Future studies will need to delineate the full window of new neuron susceptibility in females and determine whether a critical window for propofol effects on survival exists in male rats at an earlier time point.

### Conclusion

The use of anesthesia is ubiquitous in both research and clinical settings. It is therefore important for future studies, especially those addressing neurogenesis, to consider sex, type of anesthetic used, and age of newly-born cells when interpreting results. Adult neurogenesis requires the coordinated timing of several independent processes, including cell proliferation, neuronal maturation, and survival. Our results indicate that different sedatives can affect different phases of neurogenesis. Inhibiting cell proliferation and increasing cell death both have the same net effect of decreasing the number of functional new neurons, possibly resulting in cognitive or emotional impairment (Cameron and Glover, 2017; Karlsson et al., 2018; Weeden et al., 2019). However, behavioral impairments resulting from these two effects would likely be observed at different time points after sedation; effects on nearly-mature neurons should have almost immediate consequences, while changes in proliferation would impact behavior only after a delay of several weeks. Changes in cell proliferation may also be less likely to produce functional impairments than effects on older neurons because the number of new neurons can be normalized during maturation via altered rates of survival (Tanapat et al., 1999, 2001; Snyder et al., 2009b). The expected duration of functional impairments due to decreases in adult neurogenesis following sedation is unclear but would depend on how long proliferation is diminished, which appear to be short-lived (Lin et al., 2013), and the age range of maturing cells susceptible to sedation-induced loss. Future studies will be needed to determine whether sedation-induced decreases in neurogenesis result in detectable cognitive and behavioral changes, both in experimental models and in human patients.

## Data Availability Statement

The raw data supporting the conclusions of this article will be made available by the authors, without undue reservation, to any qualified researcher.

## Ethics Statement

The animal study was reviewed and approved by Animal Care and Use Committee of the National Institute of Mental Health.

## Author Contributions

JK planned the study, designed and conducted experiments, carried out data and statistical analyses, and wrote the initial draft of the manuscript. NB conducted experiments, carried out data collection, and reviewed and edited the manuscript. HC planned and designed the study and revised the manuscript. All authors discussed the results and contributed to the scientific interpretations.

## Conflict of Interest

The authors declare that the research was conducted in the absence of any commercial or financial relationships that could be construed as a potential conflict of interest.

## References

[B1] Barha C. K. Brummelte S. Lieblich S. E. Galea L. A. M. (2011). Chronic restraint stress in adolescence differentially influences hypothalamic-pituitary-adrenal axis function and adult hippocampal neurogenesis in male and female rats. *Hippocampus* 21 1216–1227. 10.1002/hipo.20829 20665592

[B2] Barker J. M. Wojtowicz J. M. Boonstra R. (2005). Where’s my dinner? Adult neurogenesis in free-living food-storing rodents. *Genes Brain Behav.* 4 89–98. 10.1111/j.1601-183X.2004.00097.x 15720405

[B3] Bayer S. A. Altman J. Russo R. J. Zhang X. (1993). Timetables of neurogenesis in the human brain based on experimentally determined patterns in the rat. *Neurotoxicology* 14 83–144. 8361683

[B4] Beery A. K. Zucker I. (2011). Neuroscience and biobehavioral reviews sex bias in neuroscience and biomedical research. *Neurosci. Biobehav. Rev.* 35 565–572. 10.1016/j.neubiorev.2010.07.002 20620164 PMC3008499

[B5] Broad K. D. Hassell J. Fleiss B. Kawano G. Ezzati M. Rocha-Ferreira E. (2016). Isoflurane exposure induces cell death, microglial activation and modifies the expression of genes supporting neurodevelopment and cognitive function in the male newborn piglet brain. *PLoS One* 11:e0166784. 10.1371/journal.pone.0166784 27898690 PMC5127656

[B6] Cabrera O. H. Gulvezan T. Symmes B. Quillinan N. Jevtovic-Todorovic V. (2020). Sex differences in neurodevelopmental abnormalities caused by early-life anaesthesia exposure: a narrative review. *Br. J. Anaesth.* 124 e81–e91. 10.1016/j.bja.2019.12.032 31980157 PMC7050624

[B7] Cahill L. Aswad D. (2015). Sex influences on the brain: an issue whose time has come. *Neuron* 88 1084–1085. 10.1016/j.neuron.2015.11.021 26687218

[B8] Callaway J. K. Wood C. Jenkins T. A. Royse A. G. Royse C. F. (2016). Isoflurane in the presence or absence of surgery increases hippocampal cytokines associated with memory deficits and responses to brain injury in rats. *Behav. Brain Res.* 303 44–52. 10.1016/j.bbr.2016.01.032 26784560

[B9] Cameron H. A. Glover L. R. (2017). Adult neurogenesis: beyond learning and memory. *Annu. Rev. Psychol.* 66 53–81. 10.1146/annurev-psych-010814-015006.ADULT PMC5612417 25251485

[B10] Cao L. Jiao X. Zuzga D. S. Liu Y. Fong D. M. Young D. (2004). VEGF links hippocampal activity with neurogenesis, learning and memory. *Nat. Genet.* 36 827–835. 10.1038/ng1395 15258583

[B11] Culley D. J. Baxter M. Yukhananov R. Crosby G. (2003). The memory effects of general anesthesia persist for weeks in young and aged rats. *Anesth. Analg.* 96 1004–1009. 10.1213/01.ANE.0000052712.67573.12 12651650

[B12] Culley D. J. Baxter M. G. Crosby C. A. Yukhananov R. Crosby G. (2004a). Impaired acquisition of spatial memory 2 weeks after isoflurane and isoflurane-nitrous oxide anesthesia in aged rats. *Anesth. Analg.* 99 1393–1397. 10.1213/01.ANE.0000135408.14319.CC 15502036

[B13] Culley D. J. Baxter M. G. Yukhananov R. Crosby G. (2004b). Long-term impairment of acquisition of a spatial memory task following isoflurane-nitrous oxide anesthesia in rats. *Anesthesiology* 100 309–314. 14739805 10.1097/00000542-200402000-00020

[B14] Culley D. J. Xie Z. Crosby G. (2007). General anesthetic-induced neurotoxicity: an emerging problem for the young and old? *Curr. Opin. Anaesthesiol.* 20 408–413. 10.1097/ACO.0b013e3282efd18b 17873593

[B15] Dayer A. G. Ford A. A. Cleaver K. M. Yassaee M. Cameron H. A. (2003). Short-term and long-term survival of new neurons in the rat dentate gyrus. *J. Comp. Neurol.* 460 563–572. 10.1002/cne.10675 12717714

[B16] Deng M. Hofacer R. D. Jiang C. Joseph B. Hughes E. A. Jia B. (2014). Brain regional vulnerability to anaesthesia-induced neuroapoptosis shifts with age at exposure and extends into adulthood for some regions. *Br. J. Anaesth.* 113 443–451. 10.1093/bja/aet469 24431386 PMC4148607

[B17] Erasso D. M. Camporesi E. M. Mangar D. Saporta S. (2013). Effects of isoflurane or propofol on postnatal hippocampal neurogenesis in young and aged rats. *Brain Res.* 1530 1–12. 10.1016/j.brainres.2013.07.035 23891717

[B18] Erasso D. M. Chaparro R. E. Quiroga del Rio C. E. Karlnoski R. Camporesi E. M. Saporta S. (2011). Quantitative assessment of new cell proliferation in the dentate gyrus and learning after isoflurane or propofol anesthesia in young and aged rats. *Brain Res.* 1441 38–46. 10.1016/j.brainres.2011.11.025 22297171

[B19] Galea L. A. M. McEwen B. S. (1999). Sex and seasonal differences in the rate of cell proliferation in the dentate gyrus of adult wild meadow voles. *Neuroscience* 89 955–964. 10199627 10.1016/s0306-4522(98)00345-5

[B20] Garcia P. Kolesky S. Jenkins A. (2010). General anesthetic actions on GABAA receptors. *Curr. Neuropharmacol.* 8 2–9. 10.2174/157015910790909502 20808541 PMC2866459

[B21] Garrity A. G. Botta S. Lazar S. B. Swor E. Vanini G. Baghdoyan H. A. (2014). Dexmedetomidine-induced sedation does not mimic the neurobehavioral phenotypes of sleep in Sprague Dawley rat. *Sleep* 38 73–84. 10.5665/sleep.4328 25325438 PMC4262959

[B22] Ge S. Sailor K. A. Ming G. L. Song H. (2008). Synaptic integration and plasticity of new neurons in the adult hippocampus. *J. Physiol.* 586 3759–3765. 10.1113/jphysiol.2008.155655 18499723 PMC2538931

[B23] Gertler R. Brown H. C. Mitchell D. H. Silvius E. N. (2001). Dexmedetomidine: a novel sedative-analgesic agent. *Proc. Bayl. Univ. Med. Cent.* 14 13–21. 16369581 10.1080/08998280.2001.11927725 PMC1291306

[B24] Giri P. K. Lu Y. Lei S. Li W. Zheng J. Lu H. (2018). Pretreatment with minocycline improves neurogenesis and behavior performance after midazolam exposure in neonatal rats. *Neuroreport* 29 153–159. 10.1097/WNR.0000000000000937 29256977 PMC5802258

[B25] Glasper E. R. Schoenfeld T. J. Gould E. (2012). Adult neurogenesis: optimizing hippocampal function to suit the environment. *Behav. Brain Res.* 227 380–383. 10.1016/j.bbr.2011.05.013 21624398

[B26] Gonçalves J. T. Schafer S. T. Gage F. H. (2016). Adult neurogenesis in the hippocampus: from stem cells to behavior. *Cell* 167 897–914. 10.1016/j.cell.2016.10.021 27814520

[B27] Harley C. W. (2007). Norepinephrine and the dentate gyrus. *Prog. Brain Res.* 163 299–318. 17765726 10.1016/S0079-6123(07)63018-0

[B28] Hill A. S. Sahay A. Hen R. (2015). Increasing adult hippocampal neurogenesis is sufficient to reduce anxiety and depression-like behaviors. *Neurop* 40 2368–2378. 10.1038/npp.2015.85 25833129 PMC4538351

[B29] Hofacer R. D. Deng M. Ward C. G. Joseph B. Hughes E. A. Jiang C. (2013). Cell age-specific vulnerability of neurons to anesthetic toxicity. *Ann. Neurol.* 73 695–704. 10.1002/ana.23892 23526697 PMC4667546

[B30] Hovaguimian F. Tschopp C. Beck-Schimmer B. Puhan M. (2018). Intraoperative ketamine administration to prevent delirium or postoperative cognitive dysfunction: a systematic review and meta-analysis. *Acta Anaesthesiol. Scand.* 62 1182–1193. 10.1111/aas.13168 29947091

[B31] Huang J. Jing S. Chen X. Bao X. Du Z. Li H. (2016). Propofol administration during early postnatal life suppresses hippocampal neurogenesis. *Mol. Neurobiol.* 53 1031–1044. 25577171 10.1007/s12035-014-9052-7

[B32] Jevtovic-Todorovic V. Hartman R. E. Izumi Y. Benshoff N. D. Dikranian K. Zorumski C. F. (2003). Early exposure to common anesthetic agents causes widespread neurodegeneration in the developing rat brain and persistent learning deficits. *J. Neurosci.* 23 876–882. 12574416 10.1523/JNEUROSCI.23-03-00876.2003 PMC6741934

[B33] Jiang-Xie L. F. Yin L. Zhao S. Prevosto V. Han B. X. Dzirasa K. (2019). A common neuroendocrine substrate for diverse general anesthetics and sleep. *Neuron* 102 1053–1065.e4. 10.1016/j.neuron.2019.03.033 31006556 PMC6554048

[B34] Jin K. Xie L. Kim S. H. Parmentier-Batteur S. Sun Y. Mao X. O. (2004). Defective adult neurogenesis in CB1 cannabinoid receptor knockout mice. *Mol. Pharmacol.* 66 204–208. 10.1124/mol.66.2.204 15266010

[B35] John A. Cole D. Espinueva D. Seib D. R. Cooke M. B. (2020). Adult-born hippocampal neurons undergo extended development and are morphologically distinct from neonatally-born neurons. *J. Neurosci.* 40 5740–5756. 32571837 10.1523/JNEUROSCI.1665-19.2020 PMC7380968

[B36] Karlsson R. M. Wang A. S. Sonti A. N. Cameron H. A. (2018). Adult neurogenesis affects motivation to obtain weak, but not strong, reward in operant tasks. *Hippocampus* 28 512–522. 10.1002/hipo.22950 29663595 PMC6021202

[B37] Karten Y. J. G. Jones M. A. Jeurling S. I. Cameron H. A. (2006). GABAergic signaling in young granule cells in the adult rat and mouse dentate gyrus. *Hippocampus* 16 312–320. 10.1002/hipo.20165 16435314

[B38] Kodama M. Satoh Y. Otsubo Y. Araki Y. Yonamine R. Masui K. (2011). Neonatal desflurane exposure induces more robust neuroapoptosis than do isoflurane and sevoflurane and impairs working memory. *Anesthesiology* 115 979–991. 21956042 10.1097/ALN.0b013e318234228b

[B39] Kong K. L. Willatts S. M. Prys-roberts C. (1989). Isoflurane compared with midazolam for sedation in the intensive care unit. *BMJ* 298 1277–1280. 2500195 10.1136/bmj.298.6683.1277 PMC1836531

[B40] Koutmani Y. Karalis K. P. (2015). Neural stem cells respond to stress hormones: distinguishing beneficial from detrimental stress. *Front. Physiol.* 6:77. 10.3389/fphys.2015.00077 25814957 PMC4356227

[B41] Kozareva D. A. Cryan J. F. Nolan Y. M. (2019). Born this way: hippocampal neurogenesis across the lifespan. *Aging Cell* 18:e13007. 10.1111/acel.13007 31298475 PMC6718573

[B42] Krzisch M. Sultan S. Sandell J. Demeter K. Vutskits L. Toni N. (2013). Propofol anesthesia impairs the maturation and survival of adult-born hippocampal neurons. *Anesthesiology* 118 602–610. 23314165 10.1097/ALN.0b013e3182815948

[B43] Ku B. Alvi R. S. May L. D. V. Hrubos M. W. Russell I. Stratmann G. (2010). Isoflurane does not affect brain cell death, hippocampal neurogenesis, or long-term neurocognitive outcome in aged rats. *Anesthesiology* 112 305–315. 10.1097/aln.0b013e3181ca33a1 20098132 PMC5214622

[B44] Kulkarni V. A. Jha S. Vaidya V. A. (2002). Depletion of norepinephrine decreases the proliferation, but does not influence the survival and differentiation, of granule cell progenitors in the adult rat hippocampus. *Eur. J. Neurosci.* 16 2008–2012. 10.1046/j.1460-9568.2002.02268.x 12453065

[B45] Landin J. D. Palac M. Carter J. M. Dzumaga Y. Santerre-Anderson J. L. Fernandez G. M. (2019). General anesthetic exposure in adolescent rats causes persistent maladaptations in cognitive and affective behaviors and neuroplasticity. *Neuropharmacology* 150 153–163. 10.1016/j.neuropharm.2019.03.022 30926450 PMC6918944

[B46] Lin N. Moon T. S. Stratmann G. Sall J. W. (2013). Biphasic change of progenitor proliferation in dentate gyrus after single dose of isoflurane in young adult rats. *J. Neurosurg. Anesthesiol.* 25 306–310. 10.1097/ANA.0b013e318283c3c7 23752046 PMC3682229

[B47] McQuaid K. R. Laine L. (2008). A systematic review and meta-analysis of randomized, controlled trials of moderate sedation for routine endoscopic procedures. *Gastrointest. Endosc.* 67 910–923. 10.1016/j.gie.2007.12.046 18440381

[B48] Monk T. Weldon B. Garvan C. Dede D. van der Aa M. T. Heilman K. (2008). Predictors of cognitive dysfunction after major noncardiac surgery. *Anesthesiology* 108 18–30. 18156878 10.1097/01.anes.0000296071.19434.1e

[B49] Moreno-Jiménez E. P. Flor-García M. Terreros-Roncal J. Rábano A. Cafini F. Pallas-Bazarra N. (2019). Adult hippocampal neurogenesis is abundant in neurologically healthy subjects and drops sharply in patients with Alzheimer’s disease. *Nat. Med.* 25 554–560. 10.1038/s41591-019-0375-9 30911133

[B50] Nishimoto R. Kashio M. Tominaga M. (2015). Propofol-induced pain sensation involves multiple mechanisms in sensory neurons. *Pflugers Arch. Eur. J. Physiol.* 467 2011–2020. 25301522 10.1007/s00424-014-1620-1

[B51] Olkkola K. T. Ahonen J. (2008). “Midazolam and other benzodiazepines,” in *Modern Anesthetics. Handbook of Experimental Pharmacology*, Vol. 182 eds Schüttler J. Schwilden H. (Berlin: Springer), 335–360. 10.1007/978-3-540-74806-9_16 18175099

[B52] Palanisamy A. Crosby G. Culley D. J. (2017). EARLY gestational exposure to isoflurane causes persistent cell loss in the dentate gyrus of adult male rats. *Behav. Brain Funct.* 13 10–14. 29279051 10.1186/s12993-017-0132-5 PMC5744391

[B53] Pandharipande P. Shintani A. Peterson J. Truman B. P. Wilkinson G. R. Dittus R. S. (2006). Lorazepam is an independent risk factor for transitioning to delirium in intensive care unit patients. *Anesthesiology* 104 21–26. 16394685 10.1097/00000542-200601000-00005

[B54] Patel S. Wohlfeil E. R. Rademacher D. J. Carrier E. J. Perry L. T. J. Kundu A. (2003). The general anesthetic propofol increases brain N-arachidonylethanolamine (anandamide) content and inhibits fatty acid amide hydrolase. *Br. J. Pharmacol.* 139 1005–1013. 10.1038/sj.bjp.0705334 12839875 PMC1573928

[B55] Rodrigues R. S. Ribeiro F. F. Ferreira F. Vaz S. H. Sebastião A. M. Xapelli S. (2017). Interaction between cannabinoid type 1 and type 2 receptors in the modulation of subventricular zone and dentate gyrus neurogenesis. *Front. Pharmacol.* 8:516. 10.3389/fphar.2017.00516 28848435 PMC5554396

[B56] Rundshagen I. (2014). Postoperative cognitive dysfunction. *Dtsch. Arztebl. Int.* 111 119–125. 10.3238/arztebl.2014.0119 24622758 PMC3959222

[B57] Sairanen M. Lucas G. Ernfors P. Castrén M. Castrén E. (2005). Brain-derived neurotrophic factor and antidepressant drugs have different but coordinated effects on neuronal turnover, proliferation, and survival in the adult dentate gyrus. *J. Neurosci.* 25 1089–1094. 10.1523/JNEUROSCI.3741-04.2005 15689544 PMC6725966

[B58] Scharfman H. Goodman J. Macleod A. Phani S. Antonelli C. Croll S. (2005). Increased neurogenesis and the ectopic granule cells after intrahippocampal BDNF infusion in adult rats. *Exp. Neurol.* 192 348–356. 10.1016/j.expneurol.2004.11.016 15755552

[B59] Schüttler J. Schwilden H. (2008). *Modern Anesthetics: Handbook of Experimental Pharmacology.* Berlin: Springer-Verlag.

[B60] Scott-Warren V. L. Sebastian J. (2016). Dexmedetomidine: its use in intensive care medicine and anaesthesia. *BJA Educ.* 16 242–246. 10.1093/bjaed/mkv047

[B61] Shen X. Liu Y. Xu S. Zhao Q. Guo X. Shen R. (2013). Early life exposure to sevoflurane impairs adulthood spatial memory in the rat. *Neurotoxicology* 39 45–56. 10.1016/j.neuro.2013.08.007 23994303

[B62] Snyder J. S. Choe J. S. Clifford M. A. Jeurling S. I. Hurley P. Brown A. (2009a). Adult-born hippocampal neurons are more numerous, faster maturing, and more involved in behavior in rats than in mice. *J. Neurosci.* 29 14484–14495. 10.1523/JNEUROSCI.1768-09.2009 19923282 PMC2830901

[B63] Snyder J. S. Glover L. R. Sanzone K. M. Kamhi J. F. Cameron H. A. (2009b). The effects of exercise and stress on the survival and maturation of adult-generated granule cells. *Hippocampus* 19 898–906. 10.1002/hipo.20552 19156854 PMC2755652

[B64] Soldin P. O. Mattison R. D. (2009). Sex differences in pharmacokinetics and pharmacodynamics. *Annu. Rev. Pharmacol. Toxicol.* 44 143–157. 10.1146/annurev.pharmtox.44.101802.121453 14744256

[B65] Soumier A. Carter R. M. Schoenfeld T. J. Cameron H. A. (2016). New hippocampal neurons mature rapidly in response to ketamine but are not required for its acute antidepressant effects on neophagia in rats. *eNeuro* 3:ENEURO.0116-15.2016. 10.1523/ENEURO.0116-15.2016 27066531 PMC4819285

[B66] Spritzer M. D. Panning A. W. Engelman S. M. Prince W. T. Casler A. E. Georgakas J. E. (2017). Seasonal and sex differences in cell proliferation, neurogenesis, and cell death within the dentate gyrus of adult wild-caught meadow voles. *Neuroscience* 360 155–165. 10.1016/j.neuroscience.2017.07.046 28757249

[B67] Stratmann G. Sall J. W. May L. D. V. Bell J. S. Magnusson K. R. Rau V. (2009). Isoflurane differentially affects neurogenesis and long-term neurocognitive function in 60-day-old and 7-day-old rats. *Anesthesiology* 110 834–848. 10.1097/ALN.0b013e31819c463d 19293705

[B68] Stratmann G. Sall J. W. May L. D. V. Loepke A. W. Lee M. T. (2010). Beyond anesthetic properties: the effects of isoflurane on brain cell death, neurogenesis, and long-term neurocognitive function. *Anesth. Analg.* 110 431–437. 10.1213/ane.0b013e3181af8015 25508825

[B69] Sukhotinsky I. Minert A. Soja P. Devor M. (2016). Mesopontine switch for the induction of general anesthesia by dedicated neural pathways. *Anesth. Analg.* 123 1274–1285. 10.1213/ANE.0000000000001489 27464977

[B70] Tanapat P. Hastings N. B. Reeves A. J. Gould E. (1999). Estrogen stimulates a transient increase in the number of new neurons in the dentate gyrus of the adult female rat. *J. Neurosci.* 19 5792–5801. 10.1523/jneurosci.19-14-05792.1999 10407020 PMC6783062

[B71] Tanapat P. Hastings N. B. Rydel T. A. Galea L. A. M. Gould E. (2001). Exposure to fox odor inhibits cell proliferation in the hippocampus of adult rats via an adrenal hormone-dependent mechanism. *J. Comp. Neurol.* 437 496–504. 10.1002/cne.1297 11503148

[B72] Tung A. Herrera S. Fornal C. A. Jacobs B. L. (2008). The effect of prolonged anesthesia with isoflurane, propofol, dexmedetomidine, or ketamine on neural cell proliferation in the adult rat. *Anesth. Analg.* 106 1772–1777. 10.1213/ane.0b013e31816f2004 18499608

[B73] Tzeng W. Y. Wu H. H. Wang C. Y. Chen J. C. Yu L. Cherng C. G. (2017). Sex differences in stress and group housing effects on the number of newly proliferated cells and neuroblasts in middle-aged dentate gyrus. *Front. Behav. Neurosci.* 10:249. 10.3389/fnbeh.2016.00249 28119581 PMC5220061

[B74] Valentine H. Williams W. O. Maurer K. J. (2012). Sedation or inhalant anesthesia before euthanasia with CO2 does not reduce behavioral or physiologic signs of pain and stress in mice. *J. Am. Assoc. Lab. Anim. Sci.* 51 50–57. 22330868 PMC3276966

[B75] Vanlersberghe C. Camu F. (2008). “Propofol,” in *Modern Anesthetics. Handbook of Experimental Pharmacology*, eds Schüttler J. Schwilden H. (Berlin: Springer), 227–252. 10.1007/978-3-540-74806-9_11 18175094

[B76] Vennekens R. Menigoz A. Nilius B. (2012). TRPs in the brain. *Rev. Physiol. Biochem. Pharmacol.* 163 27–65. 23184016 10.1007/112_2012_8

[B77] Vutskits L. Xie Z. (2016). Lasting impact of general anaesthesia on the brain: mechanisms and relevance. *Nat. Rev. Neurosci.* 17 705–717. 10.1038/nrn.2016.128 27752068

[B78] Weeden C. S. S. Mercurio J. C. Cameron H. A. (2019). A role for hippocampal adult neurogenesis in shifting attention toward novel stimuli. *Behav. Brain Res.* 376:112152. 10.1016/j.bbr.2019.112152 31419520 PMC6783384

[B79] Yagi S. Splinter J. E. J. Tai D. Wong S. Galea L. A. M. (2019). Sex differences in maturation and attrition rate of adult born neurons in the hippocampus of rats. *bioRxiv [Preprint]* 10.1101/726398 PMC7369314 32586842

[B80] Zhang Y. Xu Z. Wang H. Dong Y. Shi H. N. Culley D. J. (2012). Anesthetics isoflurane and desflurane differently affect mitochondrial function, learning, and memory. *Ann. Neurol.* 71 687–698. 10.1002/ana.23536 22368036 PMC3942786

[B81] Zhao S. Zhu Y. Xue R. Li Y. Lu H. Mi W. (2012). Effect of midazolam on the proliferation of neural stem cells isolated from rat hippocampus. *Neural Regen. Res.* 7 1475–1482. 10.3969/j.issn.1673-5374.2012.19.005 25657682 PMC4308778

[B82] Zhong Y. Chen J. Li L. Qin Y. Wei Y. Pan S. (2018). PKA-CREB-BDNF signaling pathway mediates propofol-induced long-term learning and memory impairment in hippocampus of rats. *Brain Res.* 1691 64–74. 10.1016/j.brainres.2018.04.022 29684336

[B83] Zhou J. Wang F. Zhang J. Li J. Ma L. Dong T. (2018). The interplay of BDNF-TrkB with NMDA receptor in propofol-induced cognition dysfunction: mechanism for the effects of propofol on cognitive function. *BMC Anesthesiol.* 18:35. 10.1186/s12871-018-0491-y 29621970 PMC5887174

[B84] Zhu C. Gao J. Karlsson N. Li Q. Zhang Y. Huang Z. (2010). Isoflurane anesthesia induced persistent, progressive memory impairment, caused a loss of neural stem cells, and reduced neurogenesis in young, but not adult, rodents. *J. Cereb. Blood Flow Metab.* 30 1017–1030. 10.1038/jcbfm.2009.274 20068576 PMC2949194

